# Supplementary material for: The use and impact of virtual reality programs supported by aromatherapy for older adults: A scoping review protocol
Source: PLoS One. 2025 Jan 9;20(1):e0316908. doi: 10.1371/journal.pone.0316908 (PMC11717273; doi:10.1371/journal.pone.0316908)
Supplement: S1 Appendix — (DOCX) [file pone.0316908.s001.docx]

**S1 Appendix.**

**Search strategy developed for MEDLINE (EBSCOhost)**

| **Search ID#** | **Search Terms** | **Results** |
| --- | --- | --- |
| S10 | S3 AND S6 AND S9 | 24 |
| S9 | S7 OR S8 | 4,768.188 |
| S8 | TI (aged OR elder* OR frail* OR geriatri* OR gerontol* OR old* OR senior OR senium OR septuagenarian OR oct?genarian* OR nonagenarian OR centenarian OR supercentenarian) OR AB (aged OR elder* OR frail* OR geriatri* OR gerontol* OR old* OR senior OR senium OR septuagenarian OR oct?genarian* OR nonagenarian OR centenarian OR supercentenarian) | 1,964,806 |
| S7 | (MH "Aged+") | 3,502,041 |
| S6 | S4 OR S5 | 26,417 |
| S5 | TI (Virtual W3 realit* OR mixed W3 realit* OR VR) OR AB (Virtual W3 realit* OR mixed W3 realit* OR VR) | 25,062 |
| S4 | (MH "Virtual Reality+") OR (MH "Virtual Reality Exposure Therapy") | 7,436 |
| S3 | S1 OR S2 | 160,009 |
| S2 | TI (aroma OR aromatherapy OR “essential oil*” OR fragran* OR odo#r* OR olfact* OR scent* OR smell* OR sensory W0 stimulation OR multisensory W0 stimulation) OR AB (aroma OR aromatherapy OR “essential oil*” OR fragran* OR odo#r* OR olfact* OR scent* OR smell* OR sensory W0 stimulation OR multisensory W0 stimulation) | 139,143 |
| S1 | (MH "Aromatherapy") OR ( (MH "Sensation") OR (MH "Smell") ) OR (MH "Odorants") | 53,644 |

Search conducted on May 20, 2024
