# Supplementary material for: The use and impact of virtual reality programs supported by aromatherapy for older adults: A scoping review protocol
Source: PLoS One. 2025 Jan 9;20(1):e0316908. doi: 10.1371/journal.pone.0316908 (PMC11717273; doi:10.1371/journal.pone.0316908)
Supplement: S2 Appendix — (DOCX) [file pone.0316908.s002.docx]

### **S2 Appendix.**

### **Data extraction instrument.**

| Articles  (countries) | Participants  (Sample size) | Study Designs | Interventions | outcome | Outcome measures | Findings |
| --- | --- | --- | --- | --- | --- | --- |
| Author, year |  |  |  |  |  |  |
| Author, year  Author, year  Author, year  Author, year  …... |  |  |  |  |  |  |
