# Supplementary material for: The use and impact of virtual reality programs supported by aromatherapy for older adults: A scoping review protocol
Source: PLoS One. 2025 Jan 9;20(1):e0316908. doi: 10.1371/journal.pone.0316908 (PMC11717273; doi:10.1371/journal.pone.0316908)
Supplement: Attachment — Submitted filename: Response to Reviewers LH.docx [file pone.0316908.s004.docx]

Reviewers' comments:

Reviewer's Responses to Questions

**Comments to the Author**

1. Does the manuscript provide a valid rationale for the proposed study, with clearly identified and justified research questions?

The research question outlined is expected to address a valid academic problem or topic and contribute to the base of knowledge in the field.

Reviewer #1: Yes

Reviewer #2: Partly

- **The introduction section provides the rationale for this scoping review, highlighting the potentials of integrating olfactory stimuli in VR programs. The main rationale of the study is to address the gap of a lack of review on VR supported by aromatherapy in older adults. To date, there has been no scoping review reported on VR programs supported by aromatherapy. We will summarize the evidence regarding VR programs with aromatherapy for older adults, including the outcome measures and outcomes**

2. Is the protocol technically sound and planned in a manner that will lead to a meaningful outcome and allow testing the stated hypotheses?

The manuscript should describe the methods in sufficient detail to prevent undisclosed flexibility in the experimental procedure or analysis pipeline, including sufficient outcome-neutral conditions (e.g. necessary controls, absence of floor or ceiling effects) to test the proposed hypotheses and a statistical power analysis where applicable. As there may be aspects of the methodology and analysis which can only be refined once the work is undertaken, authors should outline potential assumptions and explicitly describe what aspects of the proposed analyses, if any, are exploratory.

Reviewer #1: Yes

Reviewer #2: No

- **The scoping review does not involve experimental procedure, analysis pipeline, proposed hypotheses and statistical power analysis. This scoping review follows JBI methodology, and no analyses are exploratory.**

3. Is the methodology feasible and described in sufficient detail to allow the work to be replicable?


Descriptions of methods and materials in the protocol should be reported in sufficient detail for another researcher to reproduce all experiments and analyses. The protocol should describe the appropriate controls, sample size calculations, and replication needed to ensure that the data are robust and reproducible.

Reviewer #1: Yes

Reviewer #2: No

- **This manuscript is a scoping review protocol, which does not need controls, sample size calculations, and replication.**

4. Have the authors described where all data underlying the findings will be made available when the study is complete?

The [PLOS Data policy](https://journals.plos.org/plosone/s/materials-and-software-sharing) requires authors to make all data underlying the findings described in their manuscript fully available without restriction, with rare exception, at the time of publication. The data should be provided as part of the manuscript or its supporting information, or deposited to a public repository. For example, in addition to summary statistics, the data points behind means, medians and variance measures should be available. If there are restrictions on publicly sharing data—e.g. participant privacy or use of data from a third party—those must be specified.

Reviewer #1: Yes

Reviewer #2: Yes

5. Is the manuscript presented in an intelligible fashion and written in standard English?

PLOS ONE does not copyedit accepted manuscripts, so the language in submitted articles must be clear, correct, and unambiguous. Any typographical or grammatical errors should be corrected at revision, so please note any specific errors here.

Reviewer #1: Yes

Reviewer #2: No

- **This manuscript was prepared by several authors whose first language is English and has been professionally checked to ensure no typographical or grammatical errors .**

6. Review Comments to the Author

Please use the space provided to explain your answers to the questions above and, if applicable, provide comments about issues authors must address before this protocol can be accepted for publication. You may also include additional comments for the author, including concerns about research or publication ethics.

You may also provide optional suggestions and comments to authors that they might find helpful in planning their study.


(Please upload your review as an attachment if it exceeds 20,000 characters)

Reviewer #1: I have had the privilege of examining your work entitled " The use and impact of virtual reality programs supported by aromatherapy for older adults: A scoping review protocol" I acknowledge the research offered in the paper and compliment the thoroughness of your investigation into the subject topic. In general, I consider the work to be of high quality and directly applicable to the subject. I propose making some changes and providing recommendations for improvement.

1. Clarity in Terminology:
Ensure precision in the vocabulary used throughout the document. Although the majority of the information is well described, several concepts might be better understood by readers who are not very acquainted with the topic if they were given more explanation or context. Furthermore, substantiate their explanation using the most recent research findings. Please refer to the recommended readings for a more comprehensive understanding.
Nevertheless, the writers are recommended to tackle the following concerns before the ultimate submission and emphasize them in red for thorough verification.

The introductory portion is insufficiently lengthy and it is crucial to enhance its content. To enhance the quality of this research, it is crucial to include references to relevant works in both the introductory, literature review and discussion section and highlight all revisions for double checking.
Suggestions are:
1. https://doi.org/10.3389/fpsyg.2022.933974
2. https://doi.org/10.3389/fpsyg.2022.948061
3. https://doi.org/10.2147/PRBM.S441395
4. https://doi.org/10.3389/fpubh.2024.1228271
5. https://doi.org/10.2147/PRBM.S369020
6. https://doi.org/10.3389/fpubh.2023.1170645
7. https://doi.org/10.3389/fpubh.2024.1228271
The analysis should clearly articulate the influence of your findings with current research and provide a connection to the topic or question presented in the introduction. Verify assertions with substantiating facts and elucidate intricate arguments.
• Composing the conversation might be a demanding but fulfilling undertaking.
The capacity to analyze, establish connections, and integrate facts, while also producing a research output that has undergone peer review and dissemination, may be fulfilling. This is intended for the analysis and explanation of the main findings, as well as to emphasize the originality and importance of the research.
The discussion portion is very lengthy. It is necessary to differentiate the conclusion part and also write research implications.
Additionally, please outline the constraints of this research.
There are certain ambiguous and repetitive phrases.

- **The manuscript we submitted is a scoping review protocol, not a research study. We do not have findings, discussion, and conclusion so we are not sure about the comments on those parts.**
- **For terminology, we have now expanded the description on several systematic reviews to provide more content for background explanation.**
- **The literature suggested by reviewer related to student learning, social prescription, health impact of COVID-19, which do not relate to our topic “combination of VR and aromatherapy”. Below is the literature suggested by the reviewer:**
- Noor, U., Younas, M., Saleh Aldayel, H., Menhas, R., & Qingyu, X. (2022). Learning behavior, digital platforms for learning and its impact on university student's motivations and knowledge development. *Frontiers in Psychology, 13*, 933974-933974. <https://doi.org/10.3389/fpsyg.2022.933974>
- Younas, M., Noor, U., Zhou, X., Menhas, R., & Qingyu, X. (2022). COVID-19, students satisfaction about e-learning and academic achievement: Mediating analysis of online influencing factors. *Frontiers in Psychology, 13*, 948061-948061. <https://doi.org/10.3389/fpsyg.2022.948061>
- Younas, M., Dong, Y., Menhas, R., Li, X., Wang, Y., & Noor, U. (2023). Alleviating the effects of the COVID-19 pandemic on the physical, psychological health, and wellbeing of students: Coping behavior as a mediator. *Psychology Research and Behavior Management, 16*, 5255-5270. <https://doi.org/10.2147/PRBM.S441395>
- Menhas, R., Yang, L., Saqib, Z. A., Younas, M., & Saeed, M. M. (2024). Does nature-based social prescription improve mental health outcomes? A systematic review and meta-analysis. *Frontiers in Public Health, 12*, 1228271-1228271. <https://doi.org/10.3389/fpubh.2024.1228271>
- Peng, X., Menhas, R., Dai, J., & Younas, M. (2022). The COVID-19 pandemic and overall wellbeing: Mediating role of virtual reality fitness for physical-psychological health and physical activity. *Psychology Research and Behavior Management, 15*, 1741-1756. <https://doi.org/10.2147/PRBM.S369020>
- Menhas, R., Qin, L., Saqib, Z. A., & Younas, M. (2023). The association between COVID-19 preventive strategies, virtual reality exercise, use of fitness apps, physical, and psychological health: Testing a structural equation moderation model. *Frontiers in Public Health, 11*, 1170645-1170645. <https://doi.org/10.3389/fpubh.2023.1170645>
- Menhas, R., Yang, L., Saqib, Z. A., Younas, M., & Saeed, M. M. (2024). Does nature-based social prescription improve mental health outcomes? A systematic review and meta-analysis. *Frontiers in Public Health, 12*, 1228271-1228271. <https://doi.org/10.3389/fpubh.2024.1228271>

Reviewer #2: The article has scientific potential, and includes a current theme.

1. The justification is weak, and does not adequately describe the articles already published, and the authors must show the relevance of the present study compared to the studies already published.

- **Thanks and we appreciate this comment. We have now provided more description of several articles recently published (1,3,6,13,18,25) and highlighted the potentials of integrating olfactory stimuli in VR programs. The main rationale of the study is to address the gap of a lack of review on VR supported by aromatherapy in older adults. To date, there has been no scoping review reported on VR programs supported by aromatherapy. Current VR reviews do not focus on the combination of VR and aromatherapy, just focus on the impacts of VR [1,3,6,8] or the impacts of aromatherapy [13,18,25] separately, which emphasizes the research gap needs to be covered by this scoping review and further research. We will summarize the evidence regarding VR programs with aromatherapy for older adults, including the outcome measures and outcomes.**

2. I recommend re-reading the references that guide scoping reviews, and also this recently published protocol. The search strategy must be expanded and systematized, as scoping reviews seek to carry out a broad mapping of the literature.
MDJ, P, Godfrey, C, McInerney, P, Baldini Soares, C, Khalil, H, and Parker, D. Chapter 11: scoping reviews In: E Aromataris and Z Munn, editors. JBI manual for evidence synthesis. Adelaide: JBI (2020); Pollock, D, Peters, MDJ, Khalil, H, McInerney, P, Alexander, L, Tricco, AC, et al. Recommendations for the extraction, analysis, and presentation of results in scoping reviews. JBI Evid Synth. (2023) 21:520–32. doi: 10.11124/JBIES-22-00123;

Xavier PB, Silva ÍdS, Dantas THdM, Lopes RH, de Araújo AJ, de Figueirêdo RC and Uchôa SAdC (2024) Patient satisfaction and digital health in primary health care: a scoping review protocol. Front. Public Health. 12:1357688. doi: 10.3389/fpubh.2024.1357688.

- **Thanks for providing the two resources. The first reference provides valuable guidance on synthesis and the presentation of results, which is instrumental for our process. We will be following the process in reporting the results in the next paper – the scoping review results.**
- **The second reference is a published scoping review protocol with a search strategy similar to ours. We appreciate the example. MedRxiv, an online disciplinary repository that publishes preprints and is indexed by Google Scholar and Web of Science, is already within the search scope of our protocol. Regarding gray literature, we have included theses and dissertations via the ProQuest database, conference proceedings through the Web of Science database, and additional gray literature via Google Scholar. Furthermore, we have expanded our search to include the ACM Digital Library, IEEE Xplore Digital Library, and Compendex database. These databases focus on a broad range of topics in computing, engineering, and technology fields, ensuring that we capture potential literature on Virtual Reality (VR). In addition, we are collaborating with two medical librarians to refine the search strategy to ensure we capture all relevant and key articles.**

3. Why will you include studies with people under the age of 65?

- **This scoping review will only include studies which recruited older adults whose age is older than 65.**

4. In the title: "supportedby" to "supported by"

- **We checked and avoided words accidentally concatenated.**

Journal Requirements:

When submitting your revision, we need you to address these additional requirements.

1. Please ensure that your manuscript meets PLOS ONE's style requirements, including those for file naming. The PLOS ONE style templates can be found at 
https://journals.plos.org/plosone/s/file?id=wjVg/PLOSOne_formatting_sample_main_body.pdf and 
<https://journals.plos.org/plosone/s/file?id=ba62/PLOSOne_formatting_sample_title_authors_affiliations.pdf>

- The manuscript has now been checked to meet style requirements and author affilications requirement.

2. Please provide a complete Data Availability Statement in the submission form, ensuring you include all necessary access information or a reason for why you are unable to make your data freely accessible. If your research concerns only data provided within your submission, please write "All data are in the manuscript and/or supporting information files" as your Data Availability Statement.

- **Data availability statement has been added at the end of the manuscript before references section.**

3. Please review your reference list to ensure that it is complete and correct. If you have cited papers that have been retracted, please include the rationale for doing so in the manuscript text, or remove these references and replace them with relevant current references. Any changes to the reference list should be mentioned in the rebuttal letter that accompanies your revised manuscript. If you need to cite a retracted article, indicate the article’s retracted status in the References list and also include a citation and full reference for the retraction notice.

One or more of the reviewers has recommended that you cite specific previously published works. Members of the editorial team have determined that some of the works referenced are not directly related to the submitted manuscript. As such, please note that it is not necessary or expected to cite the works requested by the reviewer.

- The reference list has been reviewed now to ensure its accuracy.
