# Supplementary material for: The use and impact of virtual reality programs supported by aromatherapy for older adults: A scoping review protocol
Source: PLoS One. 2025 Jan 9;20(1):e0316908. doi: 10.1371/journal.pone.0316908 (PMC11717273; doi:10.1371/journal.pone.0316908)
Supplement: Attachment — Submitted filename: Response to comments.docx [file pone.0316908.s005.docx]

Comments from PLOS Editorial Office: We note that Reviewer 1 has recommended that you cite specific previously published works in an earlier round of revision. Members of the editorial team have determined that the works referenced are not directly related to the submitted manuscript. As such, please note that it is not necessary or expected to cite the works requested by the reviewer.

Additional Editor Comments:

Comments
1. In the abstract, try to avoid repeating words in a single sentence (outcomes) for example, ….”” “Therefore, this scoping 26 review will identify existing types of virtual reality programs supported by various forms of 27 aromatherapies and their outcomes and outcome measures on the well-being of older adults. “”

**Response**: Thanks for your comments. We have revised “outcomes” into “results.”

2. Also, it is mentioned that the review will identify outcomes and outcome measures but does not specify examples. Including 1-2 key examples of anticipated outcomes (e.g., stress reduction, cognitive benefits, etc)

**Response**: Thank you for your comments. We had already identified a relevant piece of literature before conducting this scoping review, which provided examples of anticipated outcomes such as happiness, sleep quality, perceived stress, and life satisfaction (Page 9, Lines 118-120, Reference 24). In response to your suggestion, we have also included a description of these outcomes in the data synthesis section (Page 15, Lines 241-242)."

3. The introduction covers a wide range of topics (VR, aromatherapy, and their integration) but it lacks a smoother transition between these topics. For example, the shift between the sections on VR and aromatherapy feels abrupt. I suggest adding a linking sentence to explain how these topics are interconnected.

**Response**: Thanks for your comments. We have added one linking sentence between these two topics. (Page 5, Line 63–65)

4. There should also be a statement of “whether VR has been used to improve mobility or where aromatherapy reduced anxiety in older adults.

**Response**: Thanks for your useful comments. We mentioned VR can improve physical health like mobility (Page 5, Line 50-51, references 4-6) and aromatherapy can reduce anxiety (Page 6, Line 71-72, reference 14 &15).

5. The contribution and novelty of the article is unclear. The authors should clearly state the contribtion and novelty of the article.

**Response**: Thanks for your comment. We have revised the introduction section to address the novelty and contribution of this scoping review. (Page 9, Line 123-124; 127-128)

6. The contribution part should include “why understanding the combined impact of VR and aromatherapy is essential?”

**Response**: Thanks for your comments. We added this contribution to the introduction section (Page 9, Line 128-130).

7. Also the motivation of the article is not satisfying. What are the exact mootivation of this article.

**Response**: Thank you for your comments. After updating the novelty and contribution in the introduction section, it now effectively supports the motivation for conducting this scoping review. (Page 9, Line 121-130).

8. The figures quality should be high some of them are blur.

**Response**: We have no figure for this scoping review protocol.

[Note: HTML markup is below. Please do not edit.]

Reviewers' comments:

Reviewer's Responses to Questions

**Comments to the Author**

1. Does the manuscript provide a valid rationale for the proposed study, with clearly identified and justified research questions?

The research question outlined is expected to address a valid academic problem or topic and contribute to the base of knowledge in the field.

Reviewer #1: No

Response: We apologize for the change in your comments from all 'YES' to all 'NO'; we understand that our efforts do not meet your requirements. Please refer to our response to Comment No. 6 for further clarification.

Reviewer #2: Yes

2. Is the protocol technically sound and planned in a manner that will lead to a meaningful outcome and allow testing the stated hypotheses?

The manuscript should describe the methods in sufficient detail to prevent undisclosed flexibility in the experimental procedure or analysis pipeline, including sufficient outcome-neutral conditions (e.g. necessary controls, absence of floor or ceiling effects) to test the proposed hypotheses and a statistical power analysis where applicable. As there may be aspects of the methodology and analysis which can only be refined once the work is undertaken, authors should outline potential assumptions and explicitly describe what aspects of the proposed analyses, if any, are exploratory.

Reviewer #1: No

Reviewer #2: Yes

3. Is the methodology feasible and described in sufficient detail to allow the work to be replicable?

Descriptions of methods and materials in the protocol should be reported in sufficient detail for another researcher to reproduce all experiments and analyses. The protocol should describe the appropriate controls, sample size calculations, and replication needed to ensure that the data are robust and reproducible.

Reviewer #1: No

Reviewer #2: Yes

4. Have the authors described where all data underlying the findings will be made available when the study is complete?

The [PLOS Data policy](https://track.editorialmanager.com/CL0/https:%2F%2Fjournals.plos.org%2Fplosone%2Fs%2Fmaterials-and-software-sharing/1/010f01936e5f1390-4a92d6c8-152d-4129-9577-cdd65484f255-000000/vUoI8FQAGVV91W6CIf_eh1c-aRQ-XaB22cqh5De6DW4=187) requires authors to make all data underlying the findings described in their manuscript fully available without restriction, with rare exception, at the time of publication. The data should be provided as part of the manuscript or its supporting information, or deposited to a public repository. For example, in addition to summary statistics, the data points behind means, medians and variance measures should be available. If there are restrictions on publicly sharing data—e.g. participant privacy or use of data from a third party—those must be specified.

Reviewer #1: No

Reviewer #2: Yes

5. Is the manuscript presented in an intelligible fashion and written in standard English?

PLOS ONE does not copyedit accepted manuscripts, so the language in submitted articles must be clear, correct, and unambiguous. Any typographical or grammatical errors should be corrected at revision, so please note any specific errors here.

Reviewer #1: No

Reviewer #2: Yes

6. Review Comments to the Author

Please use the space provided to explain your answers to the questions above and, if applicable, provide comments about issues authors must address before this protocol can be accepted for publication. You may also include additional comments for the author, including concerns about research or publication ethics.

You may also provide optional suggestions and comments to authors that they might find helpful in planning their study.

(Please upload your review as an attachment if it exceeds 20,000 characters)

Reviewer #1: The authors are unwilling or unable to address my concerns sufficiently to make this manuscript suitable for publication.

Response: We apologize that we cannot add findings, discussion, and conclusion in a protocol. We apologize that we cannot add nonrelevant references to this protocol. We do try our best to expand the description on several systematic reviews for sufficient background explanation. We are looking forward to your more constructive suggestions.

Reviewer #2: The authors submitted the manuscript with the corrections. The authors have worked hard to improve the methodology of the study, allowing it to be clearly replicated,

7. PLOS authors have the option to publish the peer review history of their article ([what does this mean?](https://track.editorialmanager.com/CL0/https:%2F%2Fjournals.plos.org%2Fplosone%2Fs%2Feditorial-and-peer-review-process%23loc-peer-review-history/1/010f01936e5f1390-4a92d6c8-152d-4129-9577-cdd65484f255-000000/MDQCsA6HiAg5a1nYOKEDB-WbM8KZ9U5aBwMS-psiIE4=187)). If published, this will include your full peer review and any attached files.

If you choose “no”, your identity will remain anonymous but your review may still be made public.

**Do you want your identity to be public for this peer review?** For information about this choice, including consent withdrawal, please see our [Privacy Policy](https://track.editorialmanager.com/CL0/https:%2F%2Fwww.plos.org%2Fprivacy-policy/1/010f01936e5f1390-4a92d6c8-152d-4129-9577-cdd65484f255-000000/7lOQ0YlY8GAHnyNerybP46Mpm7f6q49erKj60VfwwaU=187).

Reviewer #1: No

Reviewer #2: No
