# Supplementary material for: The use and impact of virtual reality programs supported by aromatherapy for older adults: A scoping review protocol
Source: PLoS One. 2025 Jan 9;20(1):e0316908. doi: 10.1371/journal.pone.0316908 (PMC11717273; doi:10.1371/journal.pone.0316908)
Supplement: Attachment — Submitted filename: Response to comments.docx [file pone.0316908.s006.docx]

Additional Editor Comments:
1. The author should clearly mention the contribution in bullets at the end of the Introduction Section.

Response: Thanks for your comments. The contribution in bullets is added at the end of the introduction section behind the “review question.” (Page 8, Line 139-150)

2. There should also be objectives of the study for easy understanding of readers.

Response: Thanks for your comments. The objectives are revised to be mentioned in two bullets for easy understanding of readers. (Page 7, Line 123, 125)

3. There should also be indication of each review question that mention the section where each question is addressed.

Response: Thanks for your suggestion. We added an explanation of the indication of each review question in the method section “Data synthesis” (Pages 13-14, Line 259 – 266). The revision ensures that each review question is clearly addressed, making the findings more oriented.
